# Spiny Keratoderma of Nonfamilial Nature and Without Systemic Disease in a Woman

**DOI:** 10.7759/cureus.5609

**Published:** 2019-09-09

**Authors:** Yelena Dokic, Jaime Tschen

**Affiliations:** 1 Dermatology, Baylor College of Medicine, Houston, USA; 2 Dermatology, St. Joseph Dermatopathology, Houston, USA

**Keywords:** spiny keratoderma, music box spine dermatosis, hyperkeratotic papules

## Abstract

Spiny keratoderma is a rare condition that presents as many small, firm hyperkeratotic papules on the palms and soles. This condition can be familial, typically arising in adolescence or young adulthood. However, if the condition arises later in adulthood, it is more frequently associated with internal malignancy. Therefore, a thorough workup is required to identify a potential underlying cancer. It is rare for spiny keratoderma to spontaneously arise in individuals in adulthood and yet not be associated with systemic disease, but such is the scenario for our patient.

## Introduction

Spiny keratoderma, or music box spine dermatosis, refers to a condition of the palms and soles that presents as many small, firm hyperkeratotic papules. The condition resembles the spines of an old-fashioned music box, hence the name music box spine dermatosis [1-2]. Spiny keratoderma is a rare, nonmalignant condition that has been described with numerous terms, such as punctate keratoderma [1], music box spiny dermatosis [2], palmar filiform parakeratotic hyperkeratosis [3], and punctate porokeratotic keratoderma [4]. Spiny keratoderma can be a cutaneous manifestation of systemic disease, which emphasizes its clinical importance. We present here a case of spiny keratoderma arising in a 46-year-old Hispanic female, with no family history or signs of internal malignancy.

## Case presentation

**Figure 1:**
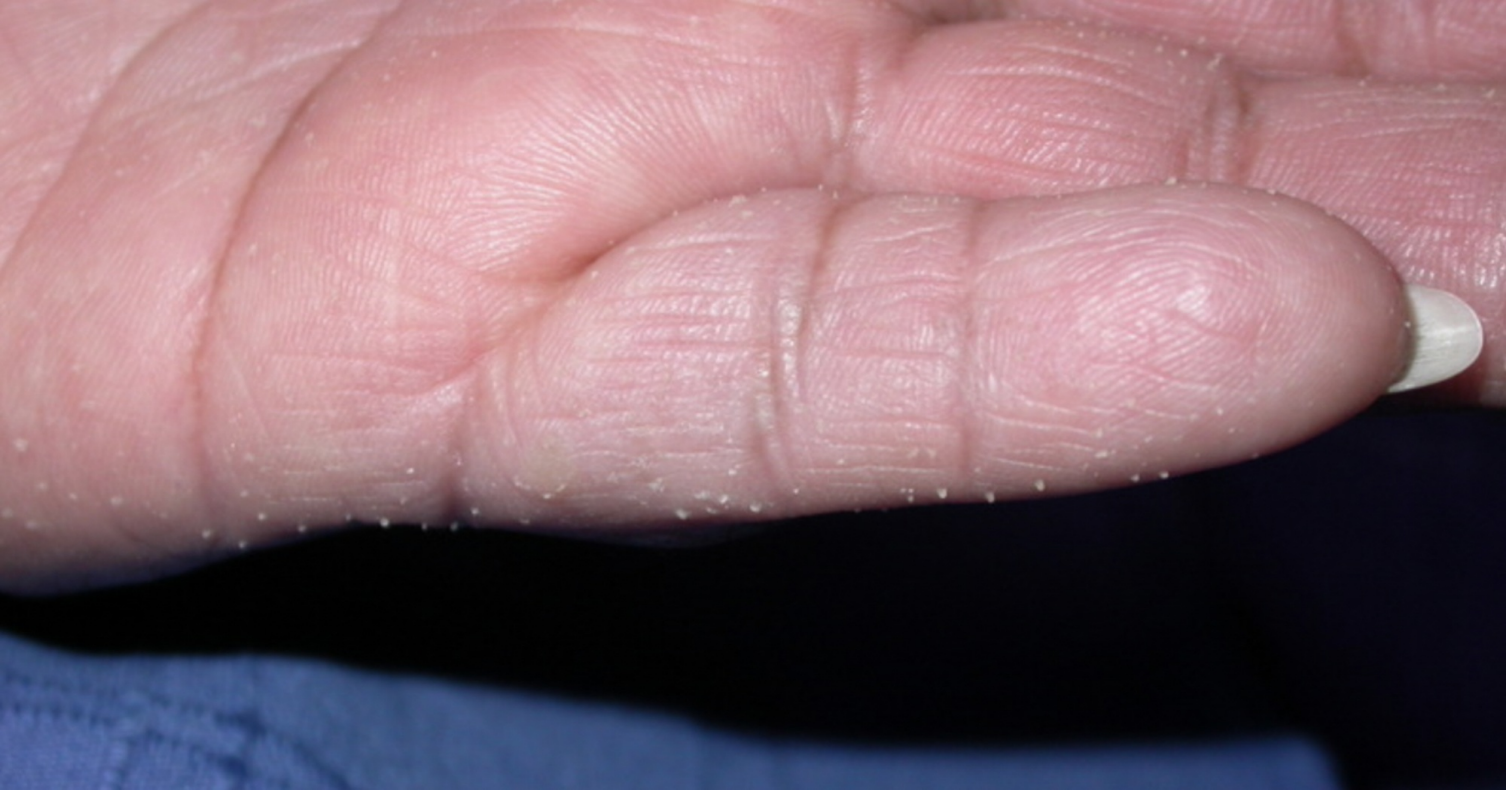
Palmar spines on the left hand. Multiple, firm one mm spicules on the volar surface of hand and digits.

A 46-year-old woman presented with multiple, small hyperkeratotic protrusions on her palms and soles (Figure 1). The papules were asymptomatic and appeared about one year ago and made her hands and feet feel dry, scaly, and crusty. She denied any family history of a similar condition. She was negative for respiratory and digestive symptoms consistent with lung or colorectal carcinoma on presentation. She was found to be vitamin D deficient. She is a nonsmoker. She denied arsenic exposure, well water consumption, or other dermatologic history. An upper gastrointestinal endoscopy was negative for any malignancies. Her current medications include ibuprofen and vitamin D. She is allergic to sulfa containing medications, which cause her to develop urticaria. Physical examination showed multiple, firm one mm spicules on the volar surface of both hands and digits. No spicules were noted on the dorsal aspects of the hands. The lesions had not received prior treatment. The remainder of the physical exam was unremarkable.

**Figure 2:**
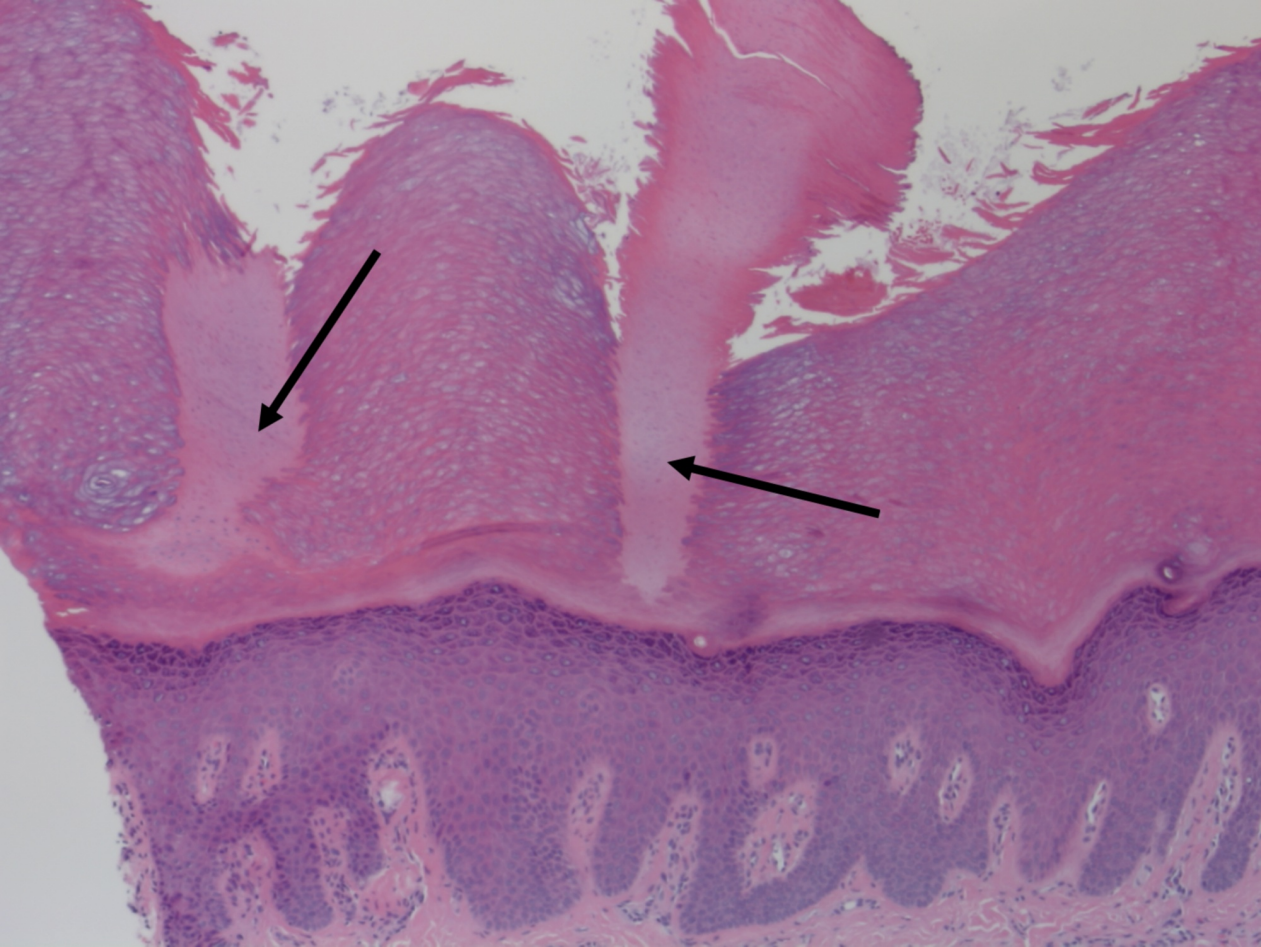
Two palmar spines. Arrows indicate palmar spines, which are columns of orthokeratotic and parakeratotic cells in stratum corneum with underlying hypogranulosis. Hematoxylin-eosin stain, original magnification 40x.

**Figure 3:**
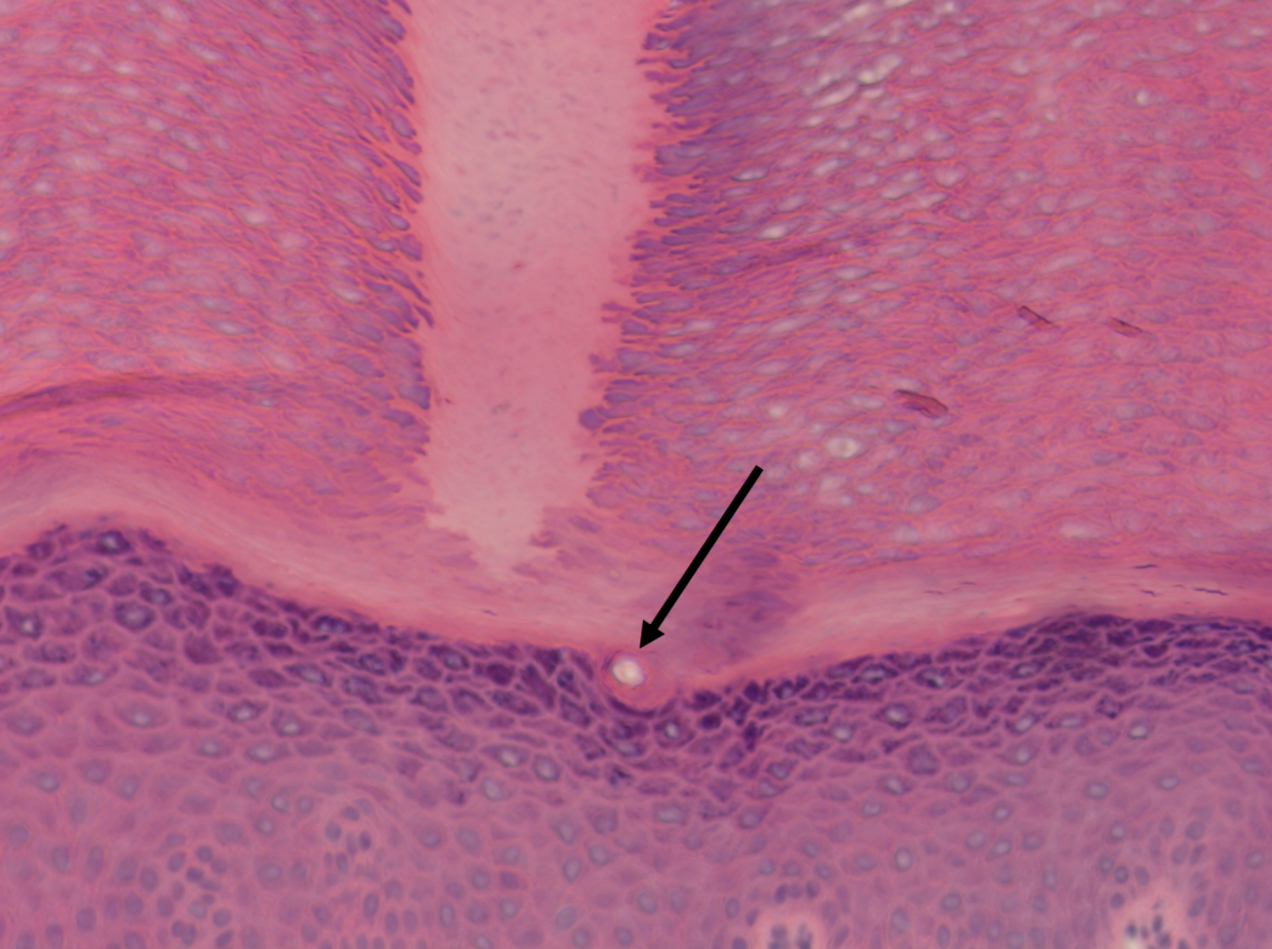
Spine arising from the epidermis without epidermal invagination, and no involvement of sweat duct. Arrow indicates sweat duct. Hematoxylin-eosin stain, original magnification 400x.

A three mm punch biopsy was performed, and histopathology revealed dense orthokeratotic keratin filling an epidermal normal base. A column of denser keratin with parakeratosis was also visualized (Figures 2-3). 

Treatment options discussed with the patient included Carmol 20, acitretin, and calcipotriene with 5-fluorouracil for one to two weeks. The patient opted for calcipotriene with 5-fluorouracil and planned for clinical monitoring.

## Discussion

Spiny keratoderma is a rare, nonmalignant condition that has been described with various terms, such as punctate keratoderma [1], music box spiny dermatosis [2], palmar filiform parakeratotic hyperkeratosis [3], and punctate porokeratotic keratoderma [4]. The first case was originally described as punctate keratoderma in 1971 by Brown [1]. Since then, only 42 reports, including ours, have been described.

Although the precise mechanism of spiny keratoderma is unknown, several proposed hypotheses exist. The interference of cholesterol synthesis by coenzyme A reductase inhibitors may disrupt lamellar bodies and result in epidermal hyperplasia [5]. Other hypotheses include repeated trauma, such as in the case of manual laborers, or ectopic hair formation [2, 5].

The patient population of spiny keratoderma follows a bimodal distribution, with a younger subset of patients typically having familial, inherited cases of the condition, and an older subset of patients having the condition associated with another systemic disease. Patients can either have both palmar and plantar involvement, or just palmar involvement. There have been no documented cases of just plantar involvement [6]. In contrast to most cases of spiny keratoderma, which occur in males older than 60 with both palmar and plantar involvement, we describe a 46-year-old woman with spiny keratoderma with palmoplantar involvement. Additionally, patients who present with spiny keratoderma at a relatively younger age, like our patient, tend to be familial in nature. However, our patient has no family history of the condition. Classically, if spiny keratoderma is familial, then it appears to be inherited in an autosomal dominant pattern. Individuals in these cases usually present between the ages of 10 and 20 [6]. It is rare for patients to present at an age in between the bimodal age distributions, without familial history or systemic disease, but that appears to be the situation for our patient.

Spiny keratoderma can be a cutaneous manifestation of systemic disease, which emphasizes its clinical importance. If acquired after age 60, it has often been known to be associated with internal malignancies such as bronchial carcinoma [4], renal cell carcinoma [7], rectal carcinoma [8], breast cancer [9], and nodular malignant melanoma [10]. Several non-neoplastic systemic diseases have also been identified to be associated with spiny keratoderma, such as type II diabetes [11], asthma [12] polycystic kidney disease [13], type IV hyperlipoproteinemia [14], and Darier’s disease [15]. Because spiny keratoderma can be associated with neoplasms or other systemic diseases, it is important for patients with these lesions to undergo routine age-appropriate health screenings and physical examinations.

Three types of palmoplantar localized digital keratoses have been classified: spiny keratoderma, arsenic keratosis, and multiple filiform verrucae [16]. However, the patient had neither reported exposure to arsenic or well water, nor were histological features of arsenic keratosis or multiple filiform verrucae visualized in our patient case. The histopathology revealed distinct columns of parakeratotic cells with a nearly absent granular layer, consistent with the diagnosis of spiny keratoderma.

Histological exam of spiny keratoderma reveals parakeratotic cells in a column in the stratum corneum with underlying hypogranulosis [16-17]. Depression of the epidermis beneath the column is observed [17]. Of note, there are no alterations in nearby blood vessels, no vacuolization or dyskeratosis, and the surrounding epidermis including the stratum corneum is normal. Electron microscopy shows a decreased number of keratohyaline granules, but normal Odland bodies. Immunohistochemical stain with AE13, which is a hair keratin, supports the hypothesis that spiny keratoderma may potentially be due to ectopic keratin formation [16]. 

There is no clearly established treatment for spiny keratoderma. Treatment for spiny keratoderma has been described as difficult and unsatisfactory [18]. However, recent reports have shown success with 0.002% topical tacalcitol [19], 5% 5-fluorouracil [17], a combination of 0.002% topical tacalcitol and 5% 5-fluorouracil [17, 19], 12% ammonium lactate [5], salicylic acid [20], or retinoids [20]. Mechanical debridement methods, such as dermabrasion or shaving the spicules off with a razor blade, have also been documented [18, 20]. Our patient was advised to start with calcipotriene and 5-fluorouracil for five days on a small area, as well as receive a chest X-ray to screen for internal malignancies.

## Conclusions

Spiny keratoderma is a rare dermatosis that onsets in a bimodal distribution of individuals younger than 20 and older than 60. Although rarer in the population between these age groups, our patient case proves that the condition may still occur between these age groups. Additionally, once the condition is identified in patients, a systemic work up is advised to monitor for systemic internal malignancies.
